# 

**Published:** 1990-12

**Authors:** 


W1 WE403

V.105 NO.4 1990

C.01---------SEQ: SR0068788

TI: WEST OF ENGLAND MEDICAL

JOURNAL 05/24/95

### WEST OF ENGLAND MEDICAL JOURNAL

**Volume 105(iv)** **December 1990**

**On the Teaching of Operative Surgery**
**Teaching and Research of the Art of Medicine**
**Bayes Theorem for the Clinician**
**Fellowship of the Royal College of General Practitioners**
**Imaging of Intracranial Tumours**
**Placental Abruption following Vaginal Administration of Prostaglandin $E_2$**
**Mummification in Ancient Egypt**
**Reports of Meetings. From our Correspondents**
**Book Reviews etc**

NATIONAL LIBRARY

JUL 0 8 1991

OF MEDICINE

FORMERLY BRISTOL MEDICO-CHIRURGICAL JOURNAL

ISSN: 0960 6440 NLM Code B5Q

